# Cooperative Multi-AP Sensing and Dynamic-Echo Suppression for Static Environment Reconstruction in Cell-Free ISAC

**DOI:** 10.3390/s26206622

**Published:** 2026-10-19

**Authors:** Xiongwei Liao, Ruoyun Liu, Xihan Zhao, Yuchen Si, Jingting Ye, Junjie Yan, Jiamin Li

**Affiliations:** School of Information Science and Engineering, Southeast University, Nanjing 211189, China; xiongwei.liao@seu.edu.cn (X.L.); ryliu@seu.edu.cn (R.L.); xihan.z@seu.edu.cn (X.Z.); yuchen_si@seu.edu.cn (Y.S.); jingtingye@seu.edu.cn (J.Y.); junjie.y@seu.edu.cn (J.Y.)

**Keywords:** cell-free ISAC, cooperative sensing, dynamic-echo suppression, static environment reconstruction

## Abstract

Distributed access points (APs) provide complementary bistatic views for cell-free integrated sensing and communication (ISAC), but moving-object echoes can obscure static surfaces in the same channel state information (CSI). We combine covariance-informed state fitting with temporal echo estimation from the observations. Their residual CSI means feed a shared multi-AP network with surface supervision and forward measurement consistency. On 24 held-out simulated geometries, the complete pipeline increased mean surface F-score from 0.446 to 0.480 and reduced squared Chamfer distance from 1.409 to 1.136 m^2^. This pipeline combines expanded training data, surface-oriented fine-tuning and phase averaging. With decoder and inference settings fixed, a controlled +10 dB moving-path test on independently ray-traced channels increased F-score from 0.256 to 0.391 through combined suppression (paired gain 0.1353; 95% interval [0.0960, 0.1729]). In the CSI-domain sensing-error test, combined input retained F-score of 0.4793 under an added velocity-error standard deviation of 8 m/s. A separate spatial-prior study increased F-score from 0.468 to 0.657 through surface refinement. On 24 independent ray-tracing test geometries, receiver adaptation yielded F-score of 0.412 versus 0.369 for a no-CSI occupancy prior (paired gain 0.0427; 95% interval [0.0108, 0.0772]). These results establish reconstruction gains from the complete pipeline and surface refinement, with a distinct suppression benefit under strong dynamic interference.

## 1. Introduction

A surface weakly observed by one access point (AP) may remain visible from another bistatic direction. Such complementary views are useful in cell-free integrated sensing and communication (ISAC), where wireless signals support both data transmission and environmental inference [1,2]. Interpreting the observations becomes harder when static surfaces and moving objects contribute to the same channel state information (CSI).

Distributed sensing combines observations from different viewpoints to recover target or scene properties. Behdad et al. studied multistatic target detection in cell-free massive MIMO using clutter statistics and power allocation subject to communication requirements [3]. Phase-synchronized multistatic imaging combines complex observations from distributed nodes to recover scene structure [4], while tensor-based processing jointly extracts channel and target parameters from structured measurements [5]. For static occupancy reconstruction, the moving objects sensed by these APs also generate interference. The sensing estimates must therefore be translated into echo components that can be separated from the static response.

Passive bistatic radar uses projection-based processing to remove direct-path and clutter components that can mask moving targets [6]. Static reconstruction instead retains the static responses and estimates the moving contributions. Position and velocity errors change the predicted delay, angle and slow-time phase, which in turn affect reconstruction from the residual CSI. Phase-coherent Wi-Fi measurements have also produced three-dimensional views of objects and emitters [7]. Although this acquisition differs from distributed AP sensing, it illustrates the role of phase consistency in radio imaging.

Reconstructing the static environment also requires a model of how the scene produces the measurements. Multi-base-station electromagnetic sensing estimates environmental properties through propagation models [8]; computational imaging examines how finite pixel size affects those models [9]. Other work combines environmental inference with additional unknowns or information sources. Joint sensing, communication and positioning couples environmental and mobile-user unknowns [10], while multi-user selection and multi-modal fusion incorporate communication and visual information [11]. Uplink–downlink cooperation combines gridding reconstruction with multi-view fusion for multi-node four-dimensional environmental reconstruction [12]. We focus on recovering static occupancy from CSI that contains both static-scene responses and moving-object echoes.

Moving-object responses carry sensing information, yet they may resemble static surfaces when retained in the reconstruction input. A location prior identifies likely contamination regions but does not specify each link’s complex echo. The residual interference also depends on velocity through phase evolution, even when the position estimate is unchanged. We use the estimated states to construct a complex echo template for each bistatic link. Position, velocity and local covariance come from multi-link association and fusion. A second estimate extracts temporal echo structure directly from the CSI. Combining the two residual means retains the geometric prior while reducing the influence of an inaccurate initial state. A shared multi-link network reconstructs occupancy and complex reflectivity with surface supervision and a forward measurement-consistency loss. We average predictions over common phase rotations to reduce dependence on an arbitrary phase origin. A separate spatial-prior study evaluates position-conditioned reconstruction.

Our contributions are the following:

- A sensing–reconstruction interface that fits dynamic echoes from estimated object states and removes them from mixed CSI before static reconstruction.
- A combination of covariance-informed state fitting and observation-derived temporal estimation that uses geometric and temporal information to recover the static CSI mean.
- A shared multi-link reconstruction model with surface supervision, forward measurement consistency and phase-averaged inference.

We evaluate how state information, AP availability and sensing errors affect reconstruction under matched conditions.

The evaluation connects each design choice to its reconstruction effect. Complete-pipeline comparisons quantify the joint benefit of training and inference, while fixed-decoder tests isolate changes in the reconstruction input. Stronger dynamic returns reveal when suppression improves occupancy, and reference-pilot calibration quantifies recovery from receiver mismatch. Separate tests assess surface refinement, measurement consistency, inference cost and adaptation to independently ray-traced channels.

## 2. System and Signal Model

### 2.1. Multi-AP Geometry and Mixed Observations

We consider synchronized transmitting APs (TAPs) at known positions $t_{i}$ and receiving APs (RAPs) at $r_{j}$. With $N_{T}$ TAPs and $N_{R}$ RAPs, calibrated arrays and separable waveforms provide $N_{T}N_{R}$ bistatic links. Moving object *k* has state $x_{k}={\left[p_{k}^{T},v_{k}^{T}\right]}^{T}$. Static geometry is represented by an occupancy probability map $O\in {\left[0,1\right]}^{N_{x}\times N_{y}\times N_{z}}$.

**Figure 1:**
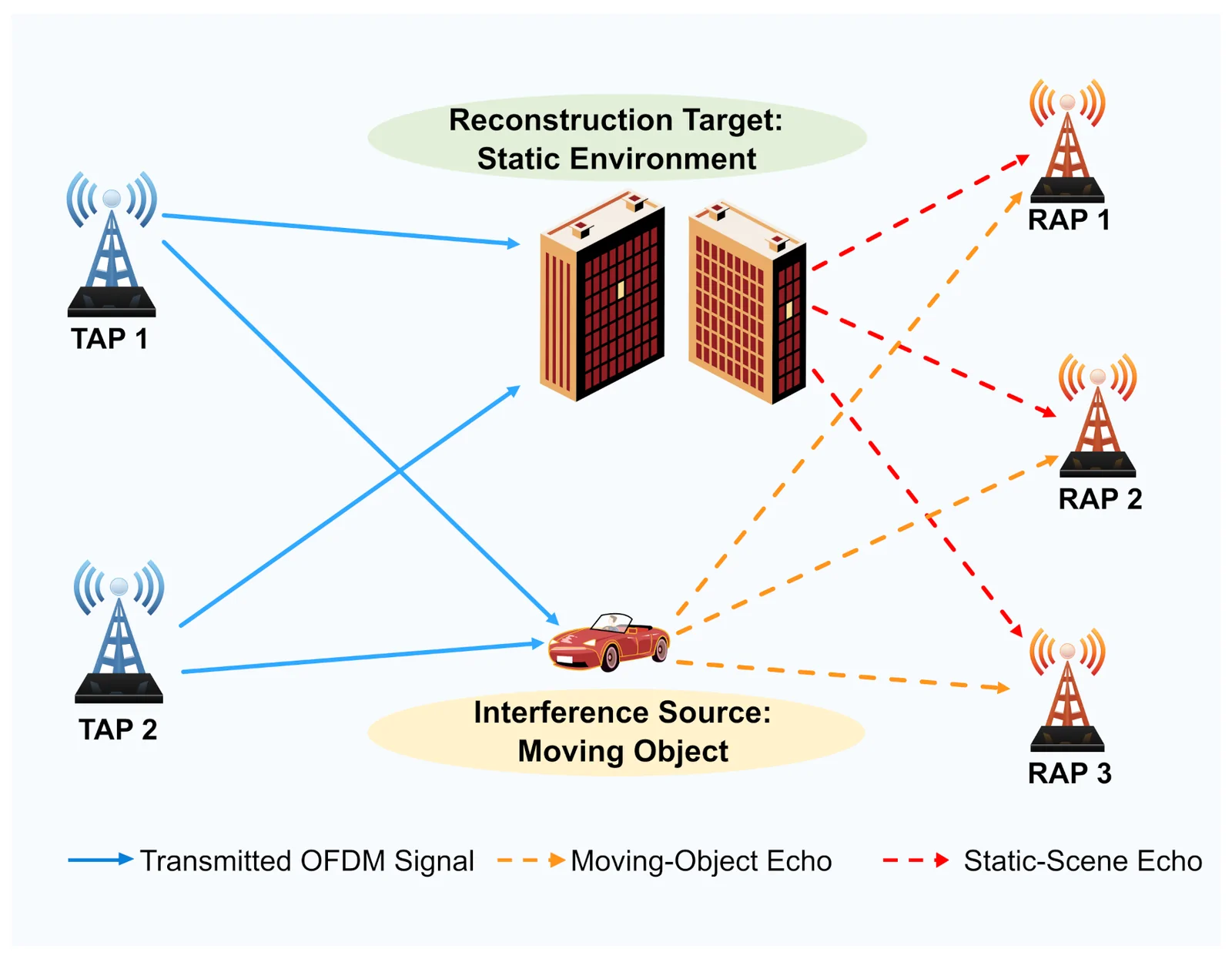
Cell-free sensing and reconstruction scenario.

In Figure 1, blue paths denote transmitted orthogonal frequency-division multiplexing (OFDM) signals, orange paths denote moving-object echoes, and red paths denote static-scene echoes. After waveform separation, link-specific CSI satisfies

(1) $$Y_{ji}=H_{ji}^{\mathrm{sta}}+H_{ji}^{\mathrm{mov}}+N_{ji},$$

where $N_{ji}$ is circular complex Gaussian noise. Let $b_{ji,\ell }\left[n\right]=\beta_{ji,\ell }e^{-j2\pi n\Delta f\tau_{ji,\ell }}$, where the unknown complex coefficient $\beta_{ji,\ell }$ includes two-segment loss, scattering, antenna gain and carrier phase. The static term is

(2) $$H_{ji}^{\mathrm{sta}}\left[n,m\right]=\sum_{\ell \in L_{\mathrm{sta}}}b_{ji,\ell }\left[n\right]a_{j,\ell }^{r}(a_{i,\ell }^{t}{)}^{H}.$$

For *K* moving objects, the time-varying term is

(3) $$H_{ji}^{\mathrm{mov}}\left[n,m\right]=\sum_{k=1}^{K}b_{ji,k}\left[n\right]e^{j2\pi mT_{s}f_{D,ji,k}}a_{j,k}^{r}(a_{i,k}^{t}{)}^{H}.$$

Here, *n* and *m* index subcarriers and slow-time samples; Δf is the subcarrier spacing and $T_{s}$ is the slow-time sampling interval. Carrier wavelength and propagation speed are $\lambda_{c}$ and *c*. The shared-CSI simulator uses receive response $e^{j2\pi d_{a}^{T}q/\lambda_{c}}$, with array-element displacement $d_{a}$ and RAP-to-object unit direction q. Transmit steering is defined through beam response ${\left(a^{t}\right)}^{H}w$, which fixes the conjugation convention in (2) and (3).

In the CSI-domain experiments, we study cooperative sensing, dynamic-echo suppression and static reconstruction from a common set of observations. Coherent sums of calibrated single-scattering responses retain geometric delays, array responses and Doppler phase evolution. Occupied surface cells carry complex scattering coefficients, and the static forward operator uses distance-dependent amplitude $800/(d_{t}d_{r})$.

In the shared-CSI experiment, successive CSI snapshots are separated by 50 μs. This slow-time interval is distinct from the OFDM useful symbol duration 1/Δf = 1 μs, which is set by the subcarrier spacing. The nominal CSI model represents synchronized, per-subcarrier observations; acquisition assumptions and residual synchronization errors are described in Section 2.4.

### 2.2. State-to-Measurement Geometry

The bistatic delay depends on both propagation segments,

(4) $$\tau_{ji,k}=\frac{∥p_{k}-t_{i}∥+∥p_{k}-r_{j}∥}{c}.$$

With unit vectors $u_{i,k}^{t}$ and $u_{j,k}^{r}$ pointing from the APs to object *k*, the Doppler frequency is

(5) $$f_{D,ji,k}=-\frac{(u_{i,k}^{t}+u_{j,k}^{r}{)}^{T}v_{k}}{\lambda_{c}}.$$

Array, frequency and slow-time samples encode direction, bistatic range ρ=cτ and range-rate $\dot{\rho }=-\lambda_{c}f_{D}$ information. Bistatic geometry relates these measurements to the object state.

### 2.3. Problem Formulation

We seek static occupancy from the mixed observation in (1). The moving objects are sensing targets in the front end and interference sources for reconstruction. The sensing interface is

(6) $${\left\{{\hat{x}}_{k},C_{x,k}\right\}}_{k=1}^{K}=F_{\mathrm{sen}}\left(\left\{Y_{ji}P\right\}\right),$$

where P removes each slow-time mean during sensing. A local covariance $C_{x,k}$ describes the fitting uncertainty. Echo suppression operates on the original CSI and returns a static mean,

(7) $${\bar{y}}_{ji}=\frac{Y_{ji}1}{N_{s}}-{\hat{h}}_{ji}^{\mathrm{mov}}\left(\hat{x},C_{x};Y_{ji}\right),$$

where ${\hat{h}}_{ji}^{\mathrm{mov}}$ combines the estimated mean dynamic contributions from state-guided and observation-derived fitting. Static reconstruction uses these residual means,

(8) $$\hat{O}=F_{\mathrm{rec}}\left(\left\{{\bar{y}}_{ji}\right\}\right).$$

Estimated velocity initializes the slow-time phase in the state-guided fit. The network receives normalized adjoint features of ${\bar{y}}_{ji}$; velocity does not enter as an additional input channel.

### 2.4. Synchronization Assumptions

The nominal observation model assumes waveform separation and AP synchronization before CSI processing. Target Doppler arises from physical path variation, whereas carrier-frequency offset (CFO) arises from residual oscillator mismatch. Both affect slow-time phase: a receiver-wide CFO can shift static and moving responses together, while object Doppler depends on bistatic geometry. Symbol timing offset (STO) can bias delay through a subcarrier-dependent phase ramp.

Residual receiver errors act on the CSI through $\exp\{j\phi_{j}-j2\pi n\Delta f\delta t_{j}+j2\pi mT_{s}\delta f_{j}\}$, where $\phi_{j}$, $\delta t_{j}$ and $\delta f_{j}$ are phase, timing and frequency offsets. The timing term introduces a subcarrier phase slope, while CFO produces a common slow-time rotation at a receiver. This approximation applies within the cyclic-prefix timing range and for frequency shifts small relative to subcarrier spacing, where intercarrier interference is weak [13]. Common-phase averaging in the reconstruction model addresses a shared phase origin; relative AP calibration is an acquisition operation.

OFDM timing and CFO can be estimated from cyclic-prefix redundancy [14]; coherent multistatic imaging also requires relative phase calibration [4]. We evaluate a receiver calibration stage that estimates RAP phase, timing and CFO from known reference packets and corrects the scene CSI before sensing. A calibrated static reference path separates receiver clock offsets from target Doppler. Section 6.5 evaluates the resulting reconstruction under joint receiver mismatch. Finite OFDM reference packets retain the within-symbol CFO rotation in the calibration test.

Each RAP estimates its receiver offsets by observing a known full-band QPSK reference packet through an independently calibrated static path. The normalized reference response is one. A packet contains 64 useful samples and a 16-sample cyclic prefix at 64 MHz, repeated at the 128 CSI times with spacing $T_{s}$ = 50 μs. The common transmission occupies 1.25/50=2.5% of airtime and serves all three RAPs. For known subcarrier symbols $P_{k}$, reference samples with timing offset τ are

(9) $$u_{\tau }\left[n\right]=\frac{1}{\sqrt{64}}\sum_{k=0}^{63}P_{k}e^{j2\pi kn/64-j2\pi k\Delta f\tau },\;r_{j}\left[m,n\right]=e^{j\phi_{j}+j2\pi \nu_{j}\left(mT_{s}+n/F_{s}\right)}u_{\tau_{j}}\left[n\right]+w_{j}\left[m,n\right],$$

where n=−16,…,63, $F_{s}=64$ MHz and $w_{j}$ is complex Gaussian noise independent of scene noise. Pilot SNR is defined per received sample and equals the scene-CSI SNR label. With $a_{\tau ,\nu }\left[m,n\right]=e^{j2\pi \nu (mT_{s}+n/F_{s})}u_{\tau }\left[n\right]$, the receiver maximizes $|\langle a_{\tau ,\nu },r_{j}\rangle {|}^{2}/{∥a_{\tau ,\nu }∥}^{2}$ over timing and CFO; the correlation angle estimates phase. Summation uses all 128 packets and 80 samples per packet. A timing grid of ±8 samples in 0.1 sample steps and CFO grid of ±200 Hz in 10 Hz steps initialize bounded Nelder–Mead refinement. Scene CSI is multiplied by $e^{-j{\hat{\phi }}_{j}+j2\pi k\Delta f{\hat{\tau }}_{j}-j2\pi {\hat{\nu }}_{j}mT_{s}}$ at subcarrier *k* and snapshot *m*, correcting receiver offsets while retaining target Doppler.

## 3. Cooperative Sensing and State Fusion

### 3.1. Direction and Range–Doppler Measurements

At RAP *j*, samples from all TAPs increase spatial snapshots without cross-RAP coherent combining. Writing each link array as $Y_{ji}\in C^{M_{r}\times N_{c}N_{s}}$, we compute

(10) $$R_{j}=\frac{1}{N_{T}N_{c}N_{s}}\sum_{i}Y_{ji}Y_{ji}^{H}+\epsilon I,$$

where $M_{r}$, $N_{c}$ and $N_{s}$ count receive elements, subcarriers and slow-time snapshots. The loading coefficient is ϵ>0, and I is the identity matrix. In the coupled study, $Y_{ji}$ here denotes the centered samples. Multiple signal classification (MUSIC) [15] and two-dimensional estimation of signal parameters via rotational invariance techniques (ESPRIT) [16] provide grid-based and grid-free direction candidates, respectively. We pair their estimates by angular distance.

The eigengap provides an empirical weight for combining the two direction estimates. For ordered eigenvalues $\lambda_{j,1}\geq \cdots \geq \lambda_{j,M_{r}}$ and known object count *K*, define $\delta_{j}=\max\left(\lambda_{j,K}/\lambda_{j,K+1}-1,0\right)$ and $\gamma_{j}=\delta_{j}/\left(\delta_{j}+0.5\right)$. We combine the paired directions as

(11) $${\hat{q}}_{j,k}=\mathrm{nrm}\;\left(\gamma_{j}{\hat{q}}_{j,k}^{\mathrm{ES}}+\left(1-\gamma_{j}\right){\hat{q}}_{j,k}^{\mathrm{MU}}\right),$$

where $\mathrm{nrm}\left(z\right)=z/{∥z∥}_{2}$, and ES and MU denote ESPRIT and MUSIC. A larger eigengap favors the ESPRIT estimate. We assess this rule against fixed fusion and the individual estimators. Direction-conditioned projection followed by a two-dimensional range–Doppler transform gives link measurements. Fourfold zero padding and three-point parabolic interpolation refine the strongest peak, whose peak-to-median power ratio $\eta_{ji,k}$ measures link quality.

### 3.2. Association and State Fusion

For measured direction ${\hat{q}}_{j,k}$ and bistatic range ${\hat{\rho }}_{ji,k}$, the RAP-to-object distance is

(12) $$s_{ji,k}=\frac{{\hat{\rho }}_{ji,k}^{2}-{∥r_{j}-t_{i}∥}^{2}}{2[{\hat{\rho }}_{ji,k}+{\left(r_{j}-t_{i}\right)}^{T}{\hat{q}}_{j,k}]}.$$

The position candidate is ${\tilde{p}}_{ji,k}=r_{j}+s_{ji,k}{\hat{q}}_{j,k}$. Association uses the candidates and measured directions. Within each RAP, detections from the first available TAP serve as anchors. Hungarian assignment minimizes $C_{ab}=d_{\theta ,ab}/\left(8{}^{\circ}\right)+∥{\tilde{p}}_{a}-{\tilde{p}}_{b}∥/\left(8\;m\right)$, combining angular and position disagreement. Across RAPs, enumeration minimizes the summed squared spread around coordinate-wise median positions. This benchmark assumes a known count of angularly resolvable objects and complete detections; association uses no ground-truth identities.

For trial state (p,v), the predicted range is $h_{ji}^{\rho }=∥p-t_{i}∥+∥p-r_{j}∥$. The predicted range rate is $h_{ji}^{\dot{\rho }}={\left(u_{t,i}+u_{r,j}\right)}^{T}v$. Normalized range, range-rate and tangent-plane direction residuals are denoted by $e^{\rho }$, $e^{\dot{\rho }}$ and $e^{q}$. Weighted least squares (WLS) estimates the six-dimensional state,

(13) $${\hat{x}}_{k}=\arg\min_{p,v}\;\sum_{i,j}w_{ji,k}\left[{\left(e_{ji,k}^{\rho }\right)}^{2}+{\left(e_{ji,k}^{\dot{\rho }}\right)}^{2}\right]+\sum_{j}w_{j,k}^{q}{∥e_{j,k}^{q}∥}_{2}^{2}.$$

We median-normalize valid qualities and clip them to [0.25,4] to obtain $w_{ji,k}$; unavailable links receive zero weight. Direction weights $w_{j,k}^{q}$ are clipped RAP-wise averages. Candidate positions and bistatic range rates initialize a bounded trust-region solver, which refines all six variables and returns the residual cost $J_{k}$.

We use the local state uncertainty to guide echo suppression. Let $G_{k}$ be the Jacobian of normalized residuals, $W_{k}$ their weights and $d_{k}$ their active scalar count. For full-rank states, we use

(14) $$C_{x,k}={\hat{s}}_{k}^{2}{\left(G_{k}^{T}W_{k}G_{k}\right)}^{-1},$$

which sets the Doppler refinement search range. With local rank $r_{k}$, we use ${\hat{s}}_{k}^{2}=\max\left\{1,J_{k}/\max\left(d_{k}-r_{k},1\right)\right\}$. Directions below a relative normal-matrix eigenvalue threshold of $10^{-10}$ receive variance of $10^{6}{\hat{s}}_{k}^{2}$. A single link has rank four, so its unidentifiable velocity directions receive this larger variance within the local approximation.

### 3.3. Sensing Evaluation

We evaluate sensing under paired scene realizations. The reference benchmark uses two TAPs and three RAPs, giving six bistatic links, with two moving objects in each scene. Each signal-to-noise ratio (SNR) in {−20,−15,−10,−5,0,5} dB has 80 paired scenes. Realizations use 64 subcarriers spaced by 1 MHz, 32 slow-time samples and 4×4 RAP arrays at 28 GHz. Methods share object states, channel tensors, noise, association and WLS. Root-mean-square error (RMSE) uses squared Euclidean position or velocity errors. These curves assess sensing after background removal, separately from mixed-CSI reconstruction.

**Figure 2:**
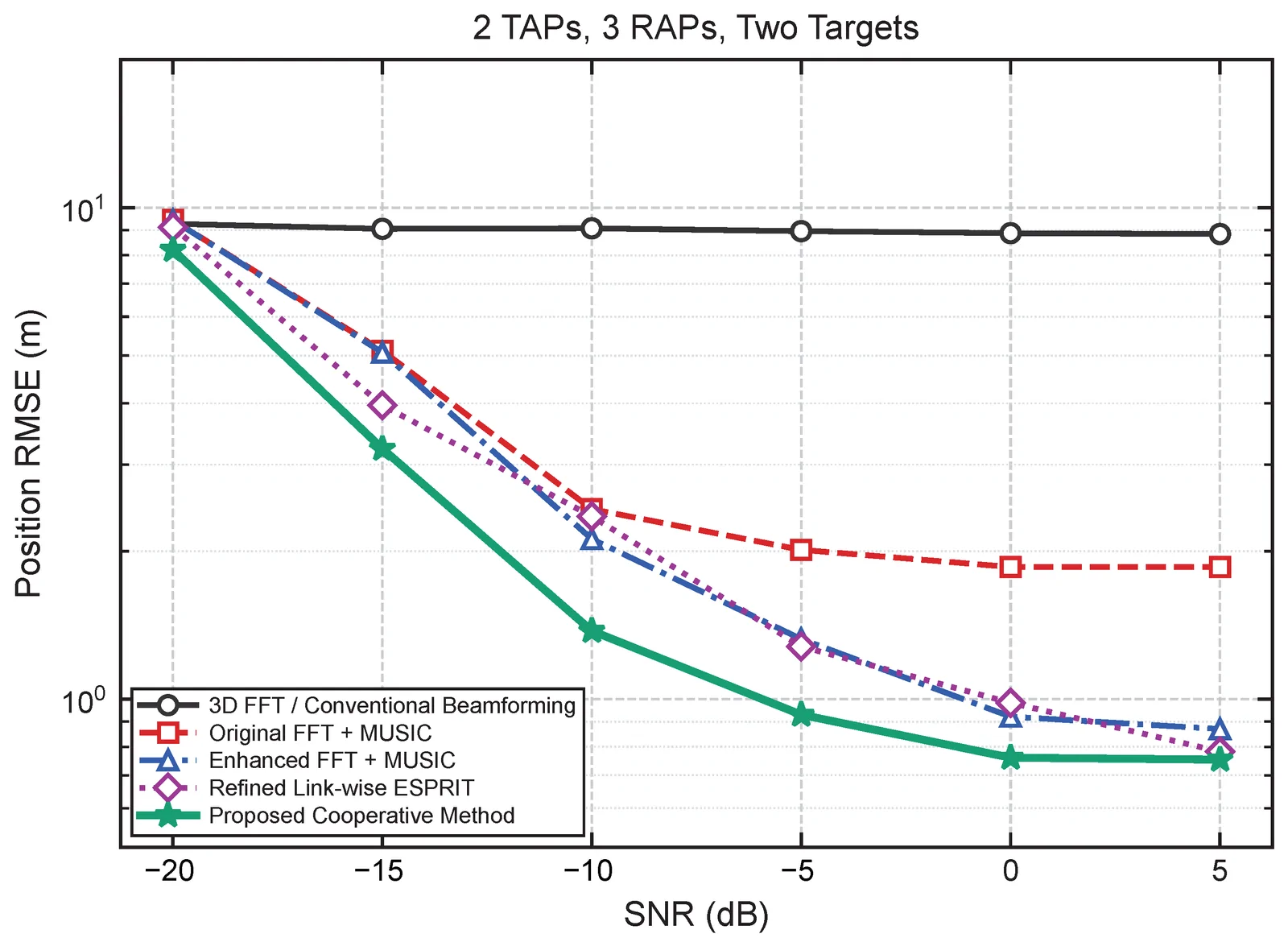
Position RMSE versus SNR; each point averages 80 paired scenes.

Figure 2 compares three-dimensional fast Fourier transform (FFT) beamforming, original and enhanced FFT–MUSIC, link-wise ESPRIT and adaptive MUSIC–ESPRIT. Across the tested SNRs, adaptive MUSIC–ESPRIT reduced position RMSE by 13.0–36.3% relative to enhanced FFT–MUSIC. Paired bootstrap difference intervals remained below zero at every SNR, including [—1.765, —0.699] m at —20 dB.

**Figure 3:**
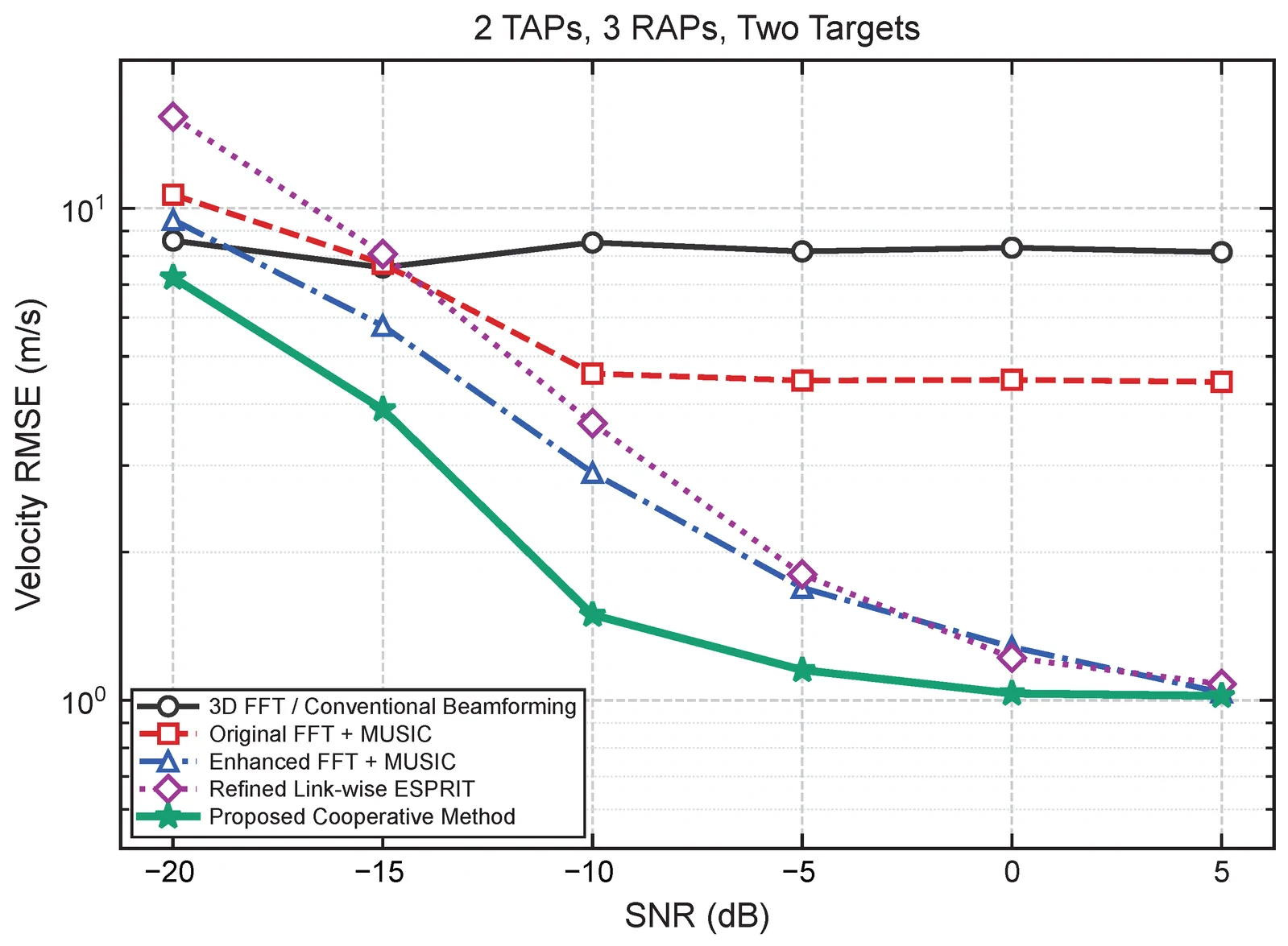
Velocity RMSE versus SNR; each point averages 80 paired scenes.

The velocity benefit varied with noise level. RMSE decreased by 1.5–48.5% in Figure 3, with difference intervals below zero from —20 to —5 dB. At 0 and 5 dB, the intervals were [—0.755, 0.002] and [—0.061, 0.007] m/s, both including zero.

We select adaptive fusion as the sensing front end on the basis of its paired sensing-benchmark gains and hold this choice fixed for reconstruction. Its downstream behavior is evaluated on the mixed observations in Section 6, with pooling, association and WLS unchanged. For MUSIC, ESPRIT, fixed-0.5 fusion and adaptive fusion, position RMSEs are 5.882, 6.401, 5.665, 5.863 m, respectively. Velocity RMSEs are 5.964, 9.482, 8.357, 5.998 m/s. Fixed fusion gives the lowest position RMSE and MUSIC the lowest velocity RMSE in these mixed observations; adaptive fusion reduces velocity RMSE relative to ESPRIT and fixed fusion.

Adaptive fusion connects sensing to dynamic-echo suppression and static reconstruction. Its state estimates initialize echo fitting for each link, while local covariances set the frequency-refinement range. The temporal structure of the observed CSI guides echo refinement, and the residual CSI is passed to the reconstruction network.

## 4. Sensing-Aided Dynamic-Echo Suppression

State-guided fitting supplies the geometry of each bistatic echo, while temporal estimation uses the echo structure observed in CSI. Combining their slow-time residual means allows reconstruction to use both sources of information before occupancy is inferred.

### 4.1. State-Guided Echo Fitting

The estimated state initializes the echo geometry: delay and Doppler follow (4) and (5), and the RAP-to-object vector determines receive direction. Let S collect each link’s array–frequency steering vectors. We stack CSI as $Y\in C^{M\times N_{s}}$, with $M=M_{r}N_{c}$, and apply ${\left(S^{H}S+10^{-3}MI\right)}^{-1}S^{H}$ to centered CSI. The regularized projection separates temporal responses for frequency refinement.

We refine the initial Doppler frequency using the projected temporal response. For object *k*, we search within half-width $2\Delta \nu +\min(2\sigma_{\nu ,k},2\Delta \nu )$, where $\Delta \nu =1/(N_{s}T_{s})$ and $\sigma_{\nu ,k}^{2}=J_{\nu ,k}C_{x,k}J_{\nu ,k}^{T}$. The Jacobian $J_{\nu ,k}$ differentiates (5) with respect to all six state coordinates. A 65-point grid and bounded scalar refinement maximize squared correlation with the projected response, normalized by centered atom energy plus $\eta N_{s}$. We skip fits with squared coherence below 0.25 or centered-to-total atom energy below 0.02. Frequencies separated by less than 0.15Δν are merged. These search, rejection and merging settings remain fixed across tests.

Let $D\in C^{N_{s}\times K^{\prime }}$ contain the retained temporal atoms and $P=I-11^{T}/N_{s}$. We jointly fit their spatial coefficients,

(15) $$\hat{B}=\arg\min_{B}∥PY^{T}{-PDB∥}_{F}^{2}+\eta {∥DB∥}_{F}^{2},$$

where validation selects η=0.1. The penalty limits fitted waveform energy when centering leaves little temporal variation. With the retained atoms fixed, one joint linear solve avoids sequential subtraction.

Free spatial coefficients accommodate steering mismatch but may absorb noise. For $\beta_{k}$, we retain its projection $\beta_{\|,k}$ onto the state steering vector and shrink the orthogonal residual $r_{k}$,

(16) $${\tilde{\beta }}_{k}=\beta_{\|,k}+{\left[1-\frac{\left(M-1\right)v_{k}}{∥r_{k}{∥}^{2}}\right]}_{+}r_{k}.$$

Here, $v_{k}$ is the fitted coefficient’s noise variance, propagated through the regularized linear solve. Noise power is estimated from the median of the lowest ten spatial-covariance eigenvalues, with a centering correction. The complete fitted waveform is subtracted,

(17) $${\tilde{Y}}^{\mathrm{state}}=Y-{\left(D\tilde{B}\right)}^{T}.$$

Centering assists dynamic estimation, while subtracting the fitted waveform from the original CSI retains the static component.

### 4.2. Temporal Estimation from the Observations

An inaccurate state can place the frequency search away from the observed echo. We also estimate temporal modes from $H_{c}=YP$, using the eigendecomposition of $H_{c}^{T}H_{c}^{*}$. At most *K* modes are retained, with eigenvalues above $1.05{\hat{\sigma }}_{n}^{2}{\left(\sqrt{M}+\sqrt{N_{s}}\right)}^{2}$. Here, ${\hat{\sigma }}_{n}^{2}$ is the observation-based noise estimate used above. If no mode passes this threshold, the measured mean is retained.

Temporal centering removes the constant response. We augment the retained eigenvectors with a constant vector, orthonormalize them and solve the one-sample shift relation as in ESPRIT [16]. The root nearest zero frequency is discarded; frequencies within 0.04Δν of zero are excluded. A joint bounded search adjusts each remaining frequency within 0.3Δν by maximizing explained centered energy. Its projection uses ridge $10^{-4}N_{s}$, with at most 35 iterations. These estimates use the measured CSI and the known object count, without requiring the initial object states.

The corresponding temporal atoms enter (15) with η=0.03 and a numerical ridge of $10^{-7}N_{s}$. Each fitted coefficient is reshaped into an $M_{r}\times N_{c}$ matrix. We retain its leading singular vectors and shrink the singular value $s_{k}$ to ${\left[s_{k}-v_{k}\left(M_{r}+N_{c}-1\right)/s_{k}\right]}_{+}$, using propagated coefficient-noise variance $v_{k}$. This rank-one form follows the separable array and subcarrier response of a single simulated path. Subtracting the fitted contribution to the temporal mean gives ${\bar{y}}^{\mathrm{obs}}$. We combine it with ${\bar{y}}^{\mathrm{state}}$ from (17),

(18) $$\bar{y}=\left(1-\alpha \right){\bar{y}}^{\mathrm{state}}+\alpha {\bar{y}}^{\mathrm{obs}},\;\alpha =0.75.$$

The development set fixes α before confirmation. Both estimates remain active: the state supplies geometric information, while the temporal estimate responds to the observed echoes.

## 5. Multi-Link Static Environment Reconstruction

### 5.1. Shared Encoding and Dual-Scale Decoding

The shared-CSI receiver maps the real part, imaginary part and magnitude of each link’s normalized adjoint CSI response to a common feature space. A shared two-layer, 12-channel encoder extracts features, which are averaged over available links. Fine and average-pooled coarse branches each use 24 channels; trilinear interpolation restores coarse features to the original grid before concatenation and decoding. The separate spatial-prior receiver described below also uses predecessor networks and attention gates.

The forward operator requires complex reflectivity to represent the scattered field’s phase. The 98,751-parameter model predicts occupancy $\hat{O}$ together with complex amplitude $\hat{c}$, with both amplitude components bounded by two. Their product $\hat{\rho }=\hat{O}⊙\hat{c}$ gives the complex reflectivity used in measurement consistency.

Occupancy supervision and measurement matching constrain different outputs of the shared network. BCE, Dice and distance losses locate the surface, while the measurement loss supervises the predicted complex field. A zero measurement coefficient removes direct field supervision of the amplitude outputs, although occupancy losses continue to update their shared features.

### 5.2. Forward Measurement Consistency

For the CSI-domain experiments, the static single-scattering operator $A_{ji}$ contains calibrated array response, bistatic phase and amplitude $800/(d_{t}d_{r})$, with grid-to-TAP/RAP distances $d_{t},d_{r}$. It generates the simulated static CSI and maps reconstructed reflectivity back to the measurement domain. For grid cell *g*, receive element *a* and subcarrier *n*, its coefficient is

(19) $$A_{ji,a,n,g}=\frac{800}{d_{t,ig}d_{r,jg}}e^{j2\pi d_{a}^{T}q_{jg}/\lambda_{c}}e^{-j2\pi \left(f_{c}+n\Delta f\right)\left(d_{t,ig}+d_{r,jg}\right)/c},$$

where $q_{jg}$ points from RAP *j* to the cell. Every occupied cell contributes coherently to each link. We minimize

(20) $$L_{\mathrm{meas}}=\frac{\sum_{ji}{∥A_{ji}\left(\hat{O}⊙\hat{c}\right)-{\bar{y}}_{ji}∥}^{2}}{\sum_{ji}{∥{\bar{y}}_{ji}∥}^{2}},$$

where ${\bar{y}}_{ji}$ is the slow-time mean of the input observation for the condition being evaluated. We use the cleaned mean for suppression and the corresponding measured mean for raw or static-only controls, summing over available links. Measurement matching constrains the complex field, while occupancy supervision specifies the surface support. During fine-tuning, we use

(21) $$L=0.5L_{\mathrm{BCE}}+1.5L_{\mathrm{Dice}}+0.25L_{\mathrm{dist}}+0.01L_{\mathrm{meas}}.$$

The positive BCE weight is five. The distance term averages ${\hat{O}}_{g}\left(1-O_{g}\right)d_{g}/\left(5\;m\right)$ over grid cells, where $d_{g}$ is the distance to the nearest true surface cell. It penalizes occupancy away from the surface. Training samples 128 array–frequency rows per link for measurement consistency; diagnostics use the full operator.

Section 6.6 evaluates how the measurement-loss coefficient affects geometric accuracy and the complex-field residual.

At inference, each observation is evaluated under 16 equally spaced common phase rotations. We average occupancy probabilities over these rotations and three trained models. Complex reflectivity is rotated back to the original phase before averaging. The threshold is fixed at 0.575. The network topology remains unchanged. Combining 16 phases and 3 models requires 48 forward passes per observation, compared with 3 for the baseline without phase averaging.

The inference-cost comparison in Section 6.6 evaluates one to sixteen phases with fixed checkpoints and threshold. All primary comparisons use the development-selected 16-phase setting.

### 5.3. Spatial-Prior Reference Receiver

**Figure 4:**
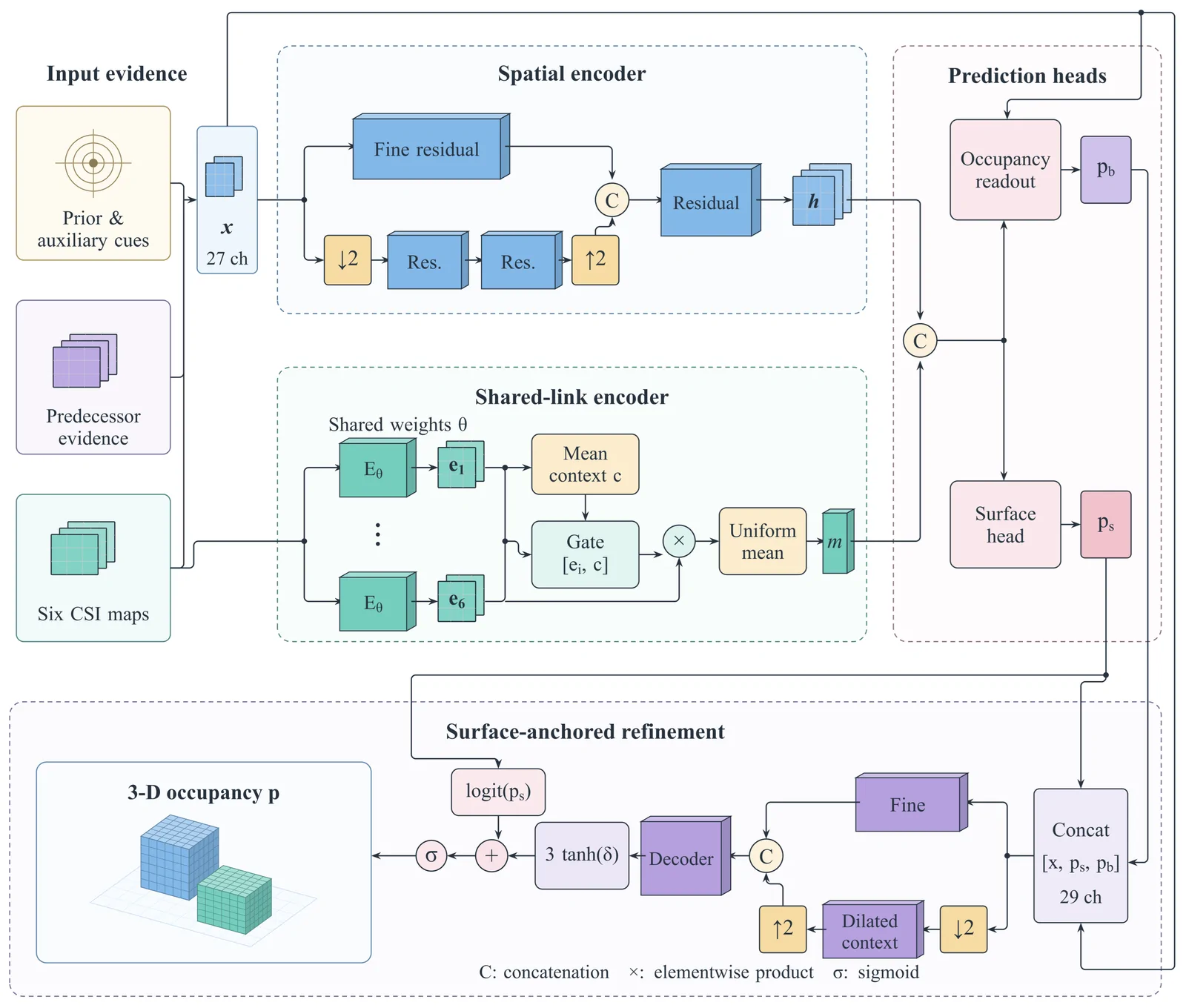
Spatial-prior receiver with surface-anchored occupancy refinement. Prior and auxiliary cues, predecessor evidence and six CSI maps form the 27-channel input. Dual-scale spatial features and gated link features feed the occupancy and surface heads. The refiner combines these outputs with the input tensor and adds a bounded correction to the surface logit. All link encoders share weights; gated link features are averaged uniformly. Blue, teal, pink and purple distinguish spatial encoding, link encoding, prediction heads and refinement modules, respectively; arrows show data flow.

The reference receiver uses estimated position and fit indicators to mark likely interference regions, without velocity or ground-truth position as inputs. Figure 4 traces the input evidence through spatial and shared-link encoders, prediction heads and surface-anchored refinement. We evaluate this spatial conditioning under the separate protocol in Section 6.

The 27 input channels contain predecessor estimates, disagreement statistics, 6 CSI responses, cross-link support, fit quality, SNR and a coarse map. The position prior is

(22) $$g_{\mathrm{mov}}\left(g\right)=\max_{k}\exp\;\left[-\frac{∥r_{g}-{\hat{p}}_{k}{∥}^{2}}{2\sigma_{k}^{2}}\right],$$

where $r_{g}$ is the grid-cell center. Its empirical width depends on WLS fit quality and is clipped to 0.65–1.55 m. Channel 23 carries this prior at scale 0.15, marking likely interference support without deleting a region. These features form the 27-channel input tensor for the dual-scale reconstruction trunk.

The dual-scale trunk processes the input tensor through fine and pooled coarse branches. Fused features drive a trust head that weights predecessor logits; bounded local and two-component corrections adjust this mixture. Each residual block uses pointwise projection, group normalization, sigmoid linear units, dropout and depthwise convolution, with an identity skip or pointwise projection when channel counts differ.

Each link’s CSI passes through a shared encoder. An attention gate uses the encoded link feature and the mean encoded context across all links; the gated features are averaged uniformly. The surface head receives the concatenated trunk and fused-link features. Base occupancy combines the predecessor mixture, bounded local and candidate terms, and a gated multi-link residual.

The output module concatenates base occupancy, surface probability and the 27 input channels. Fine and pooled coarse branches process these 29 channels; two coarse spatial convolutions use dilation factors two and one. Upsampling and joint decoding produce correction δ, giving final occupancy $p=\mathrm{sigmoid}(\mathrm{logit}\left(p_{s}\right)+3\tanh\delta )$. This surface-logit skip preserves the initial surface estimate while the correction incorporates predecessor and multi-link information.

## 6. Environment-Reconstruction Results and Discussion

### 6.1. Evaluation Design

We evaluate static reconstruction at three levels. A spatial-prior study measures the benefit of surface refinement. Shared-CSI experiments then connect cooperative sensing and dynamic-echo suppression to reconstruction, separating complete-pipeline gains from changes at the receiver input. Independent ray-tracing tests reconstruction after adaptation to material-dependent propagation and quantifies recovery as moving-path interference increases.

The CSI-domain studies reconstruct a 20×20×9.5 m region on a 24×24×10 grid. Nominal evaluation scenes contain two non-overlapping cuboid surfaces; the shape-transfer experiment uses ellipsoidal shells. Table 1 collects the radio settings and dataset partitions. In the shared-CSI simulator, occupied-cell reflectivity magnitudes are uniform on [0.8,1.2]. Each cuboid has a common random phase with cell-level Gaussian perturbations of SD 0.12 rad. Moving targets have per-link magnitudes on [0.8,1.2] and independent phases, with their aggregate field scaled to the prescribed dynamic-to-static power ratio. Position and steering remain fixed over the coherent interval while velocity sets linear phase evolution. Nominal targets are separated by more than 8 m and at least 18° at each RAP. The known target count sets the sensing subspace order and temporal rank cap; association uses measured directions and geometric candidates.

The shared-CSI training set contains single- and dual-cuboid scenes. Fine-tuning combines the 384 pretraining observations with 512 observations from 256 further geometry seeds, giving 896 observations, 448 seeds and 447 distinct occupancy masks. Two seeds yield the same single-cuboid mask with different scattering coefficients and observation conditions. Geometry IDs are disjoint across training, validation, development, test and confirmation partitions; exact-mask checks find no training-mask overlap with validation, test or confirmation. The separate stress-test and calibration-test geometries are excluded from training and selection of inference settings.

Three models with seeds 101, 202 and 303 initialize from the state-guided baseline. Pretraining uses weighted BCE, soft Dice and $0.01L_{\mathrm{meas}}$ for at most 100 epochs, with 24-epoch validation patience. AdamW uses batch size eight, weight decay $2\times 10^{-4}$ and gradient limit five, with learning rate decaying from $10^{-3}$ to $10^{-4}$. Fine-tuning uses (21) for at most 40 epochs with 20-epoch patience, retaining the batch and regularization settings and applying cosine decay from $2\times 10^{-4}$ to $2\times 10^{-5}$. Training samples raw, suppressed and static-only inputs, four link-availability patterns and a common random phase rotation. Residual augmentation retains a dynamic-response amplitude fraction uniform on [0,1.25], using the simulator’s component decomposition.

Validation selects checkpoints. Development geometries select the mixture, phase count and threshold by mean F-score across nominal states, added position error 4 m and added velocity errors 2 and 8 m/s. All choices precede confirmation evaluation. The final shared-CSI receiver uses three-model probability averaging, sixteen phases and threshold 0.575. Matched input comparisons share the observation and noise, fitted decoder, phase count and threshold. The state-guided baseline instead uses the 384-observation training set, 3-model averaging and threshold 0.60, so its comparison with the complete pipeline includes training and inference changes. The zero-measurement-loss control is independently pretrained and fine-tuned with that coefficient held at zero.

The spatial-prior protocol uses 1 moving object and 32 slow-time samples. Its 384 training samples comprise 96 single-cuboid observations and 3 frames from each of 96 dual-cuboid scenes. Surface refinement starts from the pretrained reference receiver, using $0.5L_{\mathrm{BCE}}+1.5L_{\mathrm{Dice}}+0.25L_{\mathrm{dist}}$, positive BCE weight five and a penalty on occupancy outside true surfaces. Width-16 refiners train for 50 epochs at $4\times 10^{-4}$ and then 80 at $10^{-4}$; width-24 refiners train for 100 epochs at $3\times 10^{-4}$. Both use cosine decay, AdamW, batch size eight and three seeds. Validation selects an ensemble of both widths and three seeds, averages three frames and sets threshold 0.49. Responses of at most eight connected cells are removed only when mean CSI support is below 0.35. Scores are compared within each protocol.

Surface F-score uses a 0.75 m bidirectional matching tolerance. Squared Chamfer distance symmetrically averages squared nearest-neighbor distances; IoU measures exact grid overlap. For paired method contrasts, 95% intervals use the 2.5th and 97.5th percentiles of 20,000 bootstrap resamples of geometry-level differences, averaging the 6 SNR observations within each geometry first. Intervals for condition means resample the geometry-level means. Intervals are pointwise, without multiplicity adjustment, and conditional on the fitted models; training seeds are not additional geometry replicates. SNR in the shared-CSI simulator refers to combined static and dynamic power before temporal centering, and static-only controls retain the mixed observation’s noise. Occupancy labels and surface-distance maps provide training supervision. Inference uses mixed CSI, known AP and array geometry, the forward operator, the assumed object count, and estimated states and covariances; true target states are reserved for labeled oracle controls.

### 6.2. Reconstruction Performance

**Figure 5:**
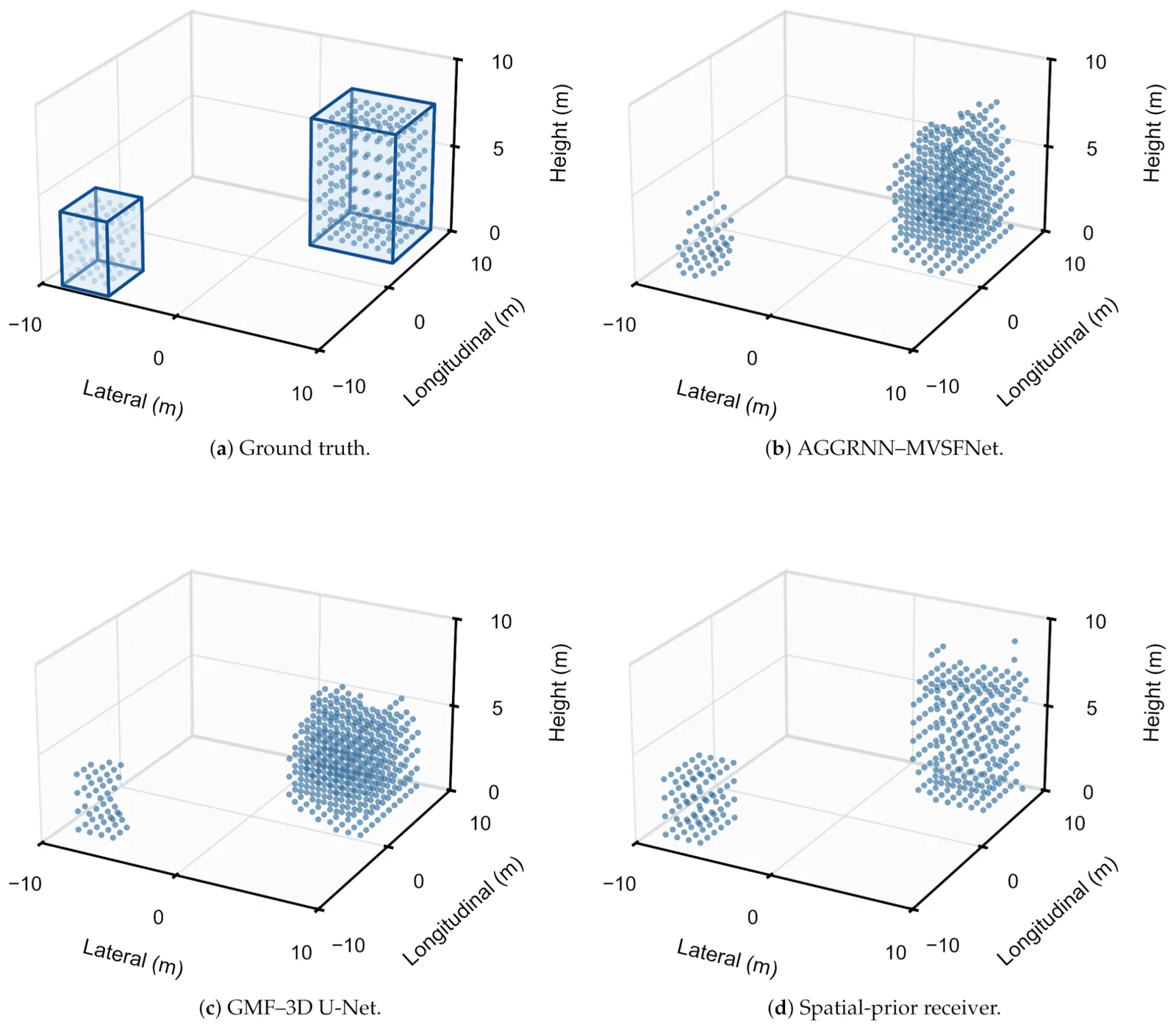
Dual-cuboid reconstruction for a fixed validation scene at 5 dB.

Surface refinement improves surface overlap and geometric accuracy in the spatial-prior study. In Table 2, F-score rises from 0.468 for the pretrained reference receiver to 0.657 after refinement, squared Chamfer distance falls from 2.721 to 0.392 m^2^, and IoU rises from 0.309 to 0.497. The paired F-score gain is 0.189 (95% CI [0.125, 0.252]). On 18 independently generated geometries from the same simulation distribution, the corresponding F-scores are 0.504 and 0.744. Figure 5 illustrates the recovered surfaces in the prespecified 5 dB validation scene, where refinement raises F-score from 0.580 to 0.851.

The coarse branch provides the largest contribution to this receiver: its removal reduces F-score by 0.341 (95% CI [0.289, 0.391]), compared with 0.087 [0.056, 0.120] for context refinement and 0.009 [0.003, 0.016] for AG enhancement. These controls freeze the checkpoints and retune thresholds on validation data only. The other architecture comparisons in Table 2 use local adaptations with their respective training protocols. AGGRNN–MVSFNet follows the gridding and multi-view fusion ideas of Lu et al. [12], with a shared attention U-Net over six fixed views and a fusion U-Net. GMF–3D U-Net combines global geometric features and per-link score volumes through a modified volumetric encoder–decoder [17]. The paired refinement and module-removal results provide comparisons within the same receiver protocol.

For the shared-CSI receiver, the complete pipeline raises confirmation F-score from 0.4464 to 0.4795, a paired gain of 0.0331 (95% CI [0.0181, 0.0482]), and reduces squared Chamfer distance from 1.409 to 1.136 m^2^ (19.4%). This comparison includes expanded training data, surface-oriented fine-tuning and phase averaging. With the final decoder fixed, raw and state-guided inputs yield confirmation F-scores of 0.4749 and 0.4780. Separating these comparisons distinguishes improvements in the full receiver from the incremental effect of suppressing dynamic echoes.

Under nominal equal-power conditions, the test-set input controls in Table 3 give similar reconstruction scores despite a 1.60 dB reduction in mean dynamic leakage relative to the raw temporal mean. Combined suppression gains 0.0021 in F-score over raw CSI (95% CI [−0.0100, 0.0123]) and 0.0016 over state-guided suppression ([−0.0032, 0.0063]); neither interval resolves a reconstruction gain. Position controls apply a mask 1−g to adjoint features. The position-only mask has Gaussian width 0.75 m, while the covariance mask uses $C_{p,k}+\mathrm{diag}\left(\Delta_{x}^{2},\Delta_{y}^{2},\Delta_{z}^{2}\right)/12$, where $C_{p,k}$ is position covariance and $\Delta_{x}$, $\Delta_{y}$, $\Delta_{z}$ are grid spacings. Oracle controls provide true position and velocity but still fit waveform coefficients from mixed CSI. Covariance-free and oracle variants retain the observation-derived estimate. Section 6.4 examines sensing errors, and Section 6.5 and Section 6.7 examine stronger dynamic interference.

### 6.3. Contributions of Sensing and Training

The shared observation provides a direct test of how sensing quality affects reconstruction. Table 4 compares five CSI inputs and four sensing front ends on twelve held-out stress-test geometries. Adaptive fusion gives the lowest mean position and velocity RMSEs, whereas downstream F-scores differ by less than 0.0006 across front ends. All adaptive-minus-alternative F-score intervals include zero. Among input procedures, state-guided fitting has the highest nominal mean F-score, whereas raw CSI has the lowest mean squared Chamfer distance; all combined-minus-control intervals include zero for the three geometric metrics. The following tests examine how reconstruction changes with sensing error and interference strength.

A 2×2×2 factorial separates the effects of training-data regime (384 or 896 observations), supervision (standard BCE–Dice or the surface objective in (21)) and inference phase count (one or sixteen). The conditions share the pretrained checkpoint within each seed and retain measurement weight 0.01. Fine-tuning follows Section 6.1, with 112 batches of 8 drawn uniformly with replacement per epoch and validation every two epochs. Validation maximizes mean F-score over three input variants and thresholds 0.2–0.8 in steps of 0.025, retaining the earlier checkpoint in a tie. Evaluation uses the 24 confirmation geometries and threshold 0.575. At sixteen phases, expanding the data increases F-score by 0.0320 (95% CI [0.0190, 0.0464]) under standard supervision and 0.0317 [0.0202, 0.0437] under surface supervision. Supervision and phase-count effect intervals include zero. The data-regime effect, which combines additional observations with residual augmentation after common pretraining, accounts for the clearest gain in this factorial comparison.

### 6.4. AP Availability and Sensing Errors

Additional AP views improve mean reconstruction scores. Across 1T1R, 1T3R, 2T3R and 2T3R with a known blocked TAP 2→RAP 3 link, suppressed inputs yield F-scores of 0.421, 0.452, 0.495 and 0.497, compared with 0.418, 0.450, 0.493 and 0.494 without suppression. The respective paired suppression gains have 95% intervals [−0.0013, 0.0081], [−0.0124, 0.0141], [−0.0100, 0.0123] and [−0.0053, 0.0118]. Unavailable links are excluded from state estimation, echo estimation and feature aggregation. Position RMSEs in the same order are 5.93, 7.76, 5.86 and 6.40 m; velocity RMSEs are 3.89, 11.11, 6.00 and 8.74 m/s. The single-link state estimate is rank deficient. AP availability changes both state estimation and the static information observed by the receiver, so sensing and reconstruction need not have the same ranking.

**Figure 6:**
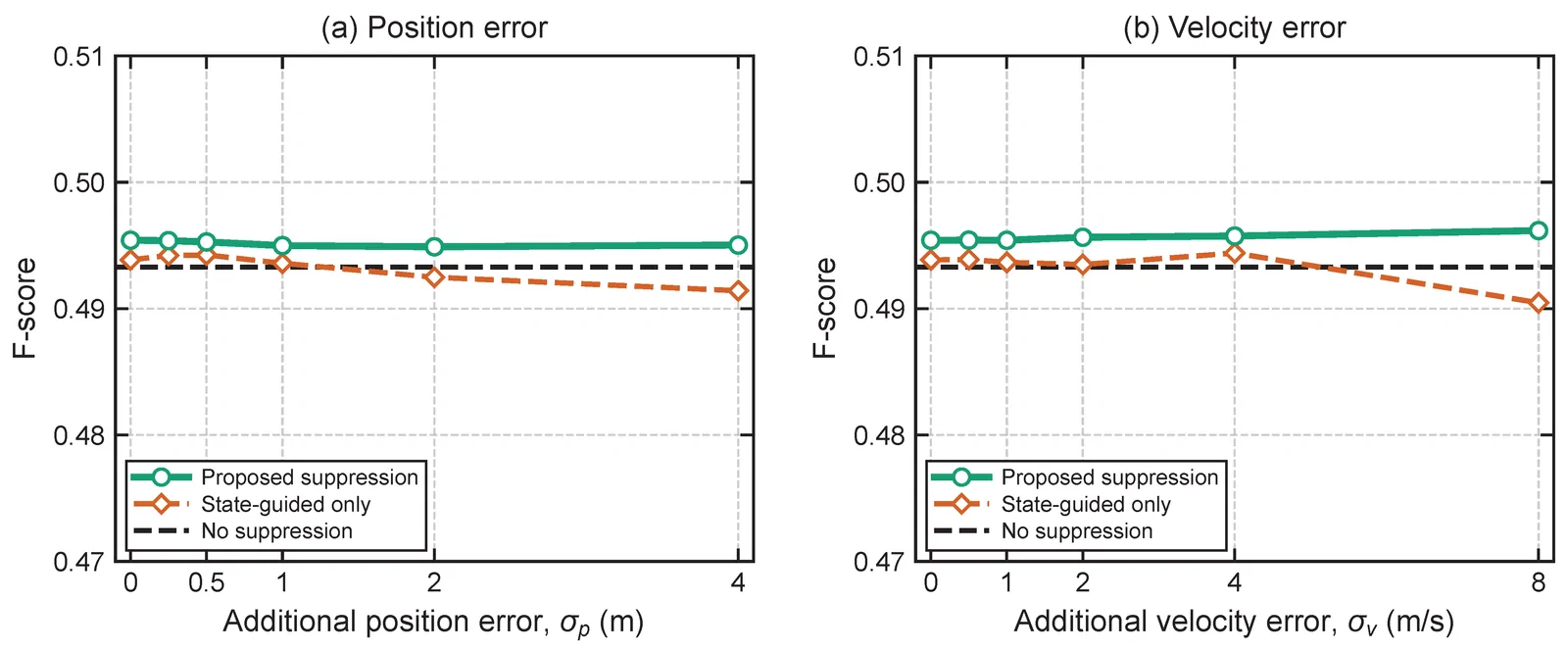
Reconstruction F-score versus added sensing error.

Figure 6 examines errors in the initial position and velocity estimates. Independent per-coordinate Gaussian perturbations use two directions per observation, reused across magnitudes. The twelve test geometries and six SNRs share observations, perturbations and receiver settings across methods; the horizontal axes report added-error SD. Combined suppression maintains F-score between 0.4949 and 0.4962, above the raw-input mean of 0.4933. At $\sigma_{p}=4$ m and $\sigma_{v}=8$ m/s, its F-scores are 0.4950 and 0.4962, compared with 0.4914 and 0.4905 for state-guided suppression. The paired gains are 0.0036 (95% CI [−0.0012, 0.0083]) and 0.0057 ([−0.0002, 0.0114]).

The confirmation set shows a similarly small change in combined-input F-score: 0.4795, 0.4794 and 0.4793 at velocity-error SDs of 0, 2 and 8 m/s. State-guided input changes from 0.4780 to 0.4763 between 0 and 8 m/s. At 8 m/s, the paired combined-minus-state-guided gain is 0.0030 (95% CI [−0.0011, 0.0079]). Across both partitions, the combined estimate maintains its mean reconstruction accuracy as the imposed state error grows, while the pairwise intervals include zero.

### 6.5. Scene Variation and Receiver Calibration

Dynamic-echo suppression is most useful when moving returns cause an appreciable reconstruction loss. Table 5a tests nineteen scene and receiver perturbations, rerunning sensing from each perturbed CSI observation with the reconstruction settings fixed. At a dynamic-to-static power ratio of +10 dB, combined suppression raises F-score from 0.4054 to 0.4539, a paired gain of 0.0485 (95% CI [0.0303, 0.0680]). At velocity scale 0.1, it gains 0.0562 [0.0437, 0.0709] over raw CSI and 0.0227 [0.0102, 0.0368] over state-guided input, although absolute accuracy is below the nominal case. At zero Doppler, the unwanted response is constant over time and all procedures give F-score of 0.2206. For these temporal fitting procedures, variation over time is essential to separating those returns from the static scene.

Power and velocity changes retain nominal noise, so their SNR labels refer to the original equal-power observation. Receiver offsets are independent zero-mean Gaussian draws per RAP, shared by its links and antennas and referenced to the first RAP; pairwise SD is $\sqrt{2}$ times the listed pre-reference SD. The delayed-path condition adds a coherent copy at amplitude −10 dB, random phase and delay uniform on 10–30 ns, with unchanged receive direction and inversion operator. Ellipsoidal shells preserve the prescribed component-power ratio and SNR.

Count tests vary the supplied subspace order and temporal rank cap, or use one or three true targets with the correct count supplied. The third target is at (0,3,8) m with velocity (0,3,0) m/s and no enforced angular separation. Target gains follow the same distribution, and total dynamic power remains equal to static power. State-list tests remove one estimate or insert a state at (5,0,6) m with velocity (3,0,0) m/s and covariance diag(4,4,4,16,16,16). A spurious state favors state-guided suppression over the combination by 0.0030 (95% CI [0.0002, 0.0065]). Timing SD 10 ns and ellipsoidal surfaces reduce combined-input F-score to 0.3461 and 0.3059. These responses distinguish sensitivity to temporal separation, state-list errors, geometric transfer and receiver alignment.

The reference-pilot estimator in Section 2.4 restores reconstruction under joint phase, timing and CFO offsets of SD 30°, 10 ns and 20 Hz. Table 5b uses twelve separate held-out calibration-test geometries at six SNRs, with settings fixed on development seeds. Ideal, uncorrected and estimated-correction conditions share scene signals, noise and receiver settings. Correction raises combined-input F-score by 0.1820 (95% CI [0.1465, 0.2177]); the corrected-minus-ideal difference is 0.0002 [−0.0012, 0.0018]. Squared Chamfer distance changes from 6.6506 m^2^ without correction to 0.9404 m^2^, close to the ideal value of 0.9345 m^2^. Raw and state-guided inputs also recover, identifying calibration as the source of this gain.

The reference transmission uses 2.5% shared airtime for all RAPs. Pilot phase, timing and CFO RMSEs fall from 9.867°, 0.597 ns and 6.004 Hz at −20 dB to 0.547°, 0.042 ns and 0.307 Hz at 5 dB. Finite OFDM evaluation gives intercarrier leakage $1.316\times 10^{-9}$ at 20 Hz CFO and 1 MHz subcarrier spacing, while phase drift accumulates across the 6.35 ms observation span. The calibration uses a known static reference path and addresses residual timing within the cyclic prefix and small CFO.

### 6.6. Measurement Supervision and Inference Cost

Forward measurement supervision improves agreement with the complex field, while surface metrics respond differently to its weight. The independently pretrained zero-loss control in Table 3 gives a normalized full-operator CSI residual of 1.004, compared with 0.532 for the retained receiver, but has higher mean F-score and IoU and lower squared Chamfer distance. Table 6a isolates the fine-tuning coefficient using common measurement-supervised initial checkpoints within each of three seeds. Coefficients 0, 0.001, 0.01 and 0.05 share 896 observations, random streams, the optimizer and the validation rule in Section 6.3.

On the confirmation set, coefficient 0.01 reduces normalized CSI residual by 0.0103 relative to zero (95% CI [0.0059, 0.0148]); its F-score difference is −0.00012 ([−0.00059, 0.00034]). Coefficient 0.05 gives the lowest mean residual in both partitions, but its F-score ranking changes between them. Individual-model F-score SDs across three seeds range from 0.0039 to 0.0047. The prespecified coefficient 0.01 is retained; these comparisons describe field agreement and occupancy under that choice.

Using a single phase substantially reduces inference cost with little change in mean reconstruction accuracy. Reducing sixteen phases to one lowers isolated decoder latency from 69.28 to 7.15 ms in Table 6b. The confirmation F-score difference, sixteen minus one phase, is 0.00012 (95% CI [−0.00265, 0.00277]). Primary comparisons retain the sixteen-phase setting selected on development data. Each model has 98,751 parameters and 0.588 billion convolution MACs per forward pass. Decoder timing uses batch size one, three warm-up calls and twenty CUDA-synchronized repetitions on an RTX 4050 Laptop GPU with PyTorch 2.6.0 and CUDA 12.6. Separate adjoint mapping takes a median 1.70 ms. These decoder measurements exclude sensing, suppression, operator construction and transfers; their peak live memory excludes operator tensors.

Timing the processing chain from mixed CSI in CPU memory to thresholded occupancy returned to CPU memory gives median latencies of 176.95 ms for one phase and 286.14 ms for sixteen phases, with interquartile ranges of 169.83–184.94 and 274.81–299.04 ms. This profile uses an Intel Core i7-14650HX with three CPU library threads and the same GPU, over nine observations from three development geometries at SNRs of −20, −10 and 5 dB, with one warm-up and five measured calls per setting. At sixteen phases, median sensing, state fitting, temporal fitting and mixing, adjoint mapping, and decoder costs are 74.50, 49.28, 47.15, 1.98 and 111.58 ms; combined transfer and thresholding take 0.43 ms. Stage medians need not sum to the median total. Peak process working set is 1503.78 MiB and peak allocated CUDA memory, including the resident operator, is 287.89 MiB. Acquisition, pilot estimation, operator construction and loading occur before the timed interval. These measurements provide a computational budget for selecting the phase count against an application’s required scene-update interval. Application-level latency, including acquisition and reference-pilot estimation, remains to be evaluated.

### 6.7. Reconstruction with Ray-Traced Channels

**Figure 7:**
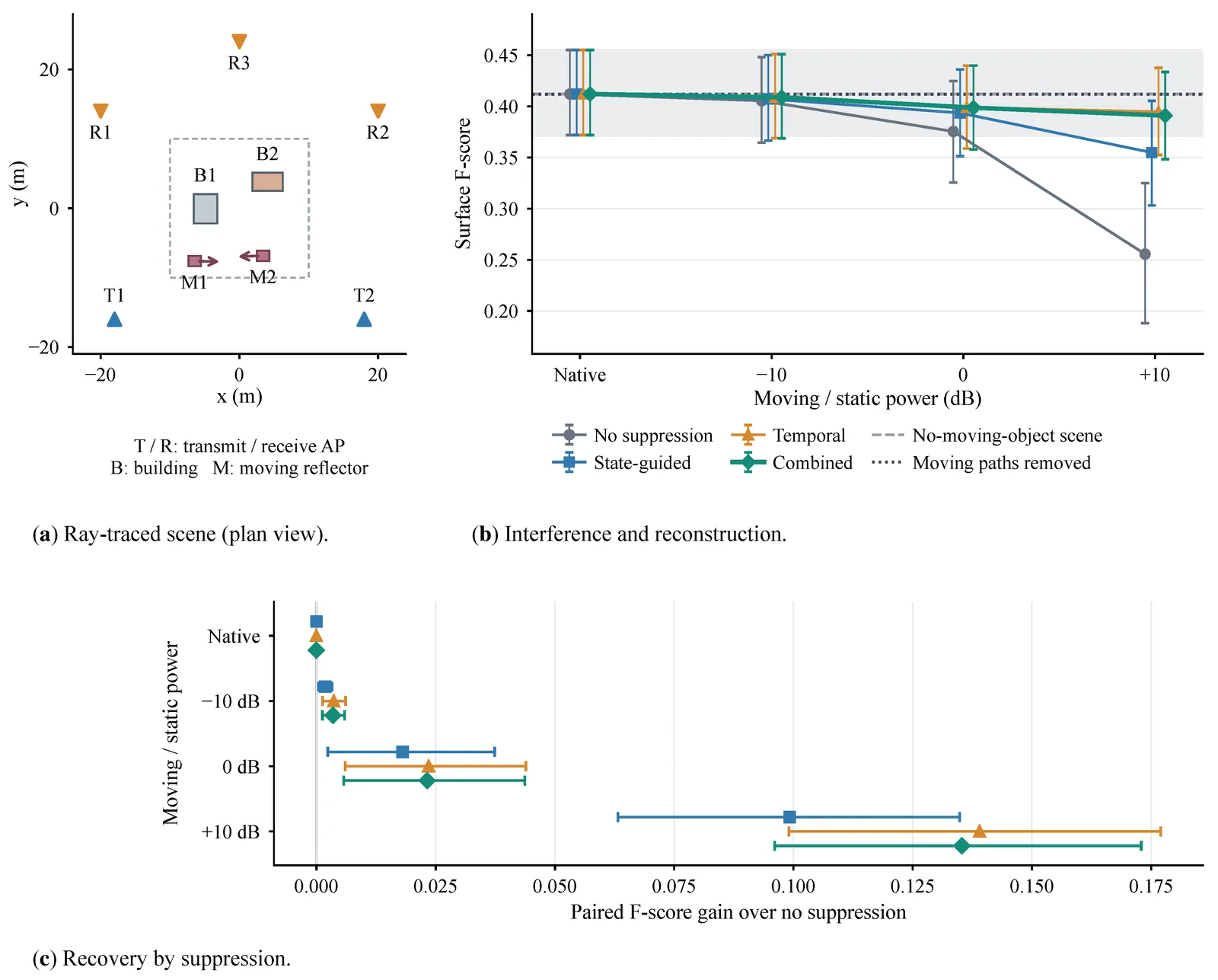
Dynamic-interference damage and recovery on ray-traced channels. (**a**) Plan view of test geometry 21030000, selected before evaluation of the controlled conditions. T and R denote transmit and receive APs; B and M denote buildings and moving metal reflectors. Arrows indicate horizontal motion directions; the dashed square bounds the reconstruction region in the horizontal plane. (**b**) Mean surface F-score across all 24 test geometries and six SNRs per geometry. Native channels are unscaled; the remaining conditions rescale only moving-path coefficients. Dashed and dotted lines denote a separately traced scene without moving objects and the diagnostic removal of moving paths from the occupied-scene solve, respectively. Error bars and reference bands are 95% geometry-bootstrap intervals. (**c**) Paired F-score gains over no suppression, with 95% intervals from 20,000 geometry-cluster resamples after averaging the six SNRs within each geometry. All methods share the adapted decoder, noise, threshold and inference settings.

Sionna RT 2.2.0 [18] provides an independent propagation model with material-dependent reflection and scattering, path visibility and multiple interactions. Each scene contains a concrete building, a brick building and two moving metal cuboids above a concrete ground plane. Table 7 collects the geometry, material and tracing parameters. Building-face intersections with the reconstruction grid define occupancy. The engine’s path coefficients, delays and Doppler frequencies generate CSI by Fourier synthesis; paths remain fixed over the 6.35 ms observation interval and evolve through their Doppler phase. Figure 7a shows one test geometry.

The dataset separates 64 training, 8 validation and 24 test geometries with disjoint seeds, each evaluated at the 6 SNRs in Section 6.1. All inputs subtract the same noiseless, phase-stable ground-only reference. This difference retains object reflections and the loss of background paths through occlusion. Separately evaluated diffuse paths in the ground-only and occupied scenes can also leave scattering–realization mismatch. Complex Gaussian noise is referenced to background-subtracted CSI power. The diagnostic static-channel mean uses non-moving paths from the occupied-scene solve, subtracts the same ground reference and averages over slow time; it is used for evaluation.

Pretraining uses the nominal single-scattering model, whereas independently ray-traced channels include material-dependent reflection and scattering, visibility changes and multiple interactions. These differences change both the amplitude and spatial structure of the reconstruction features. Receiver adaptation updates the learned feature-to-occupancy mapping for the ray-traced environment using separate training and validation geometries.

Receiver adaptation retains the shared-CSI architecture and starts from its three pretrained checkpoints. The adjoint transform is recomputed for the RT AP positions, and all four input branches use a common feature scale equal to the inverse training-set RMS of the raw-input adjoint magnitude. Each observation contributes raw, state-guided, temporal and combined inputs equally, with the same random common-phase rotation. Occupancy supervision trains the receiver for 20 epochs using AdamW with learning rate and weight decay $10^{-4}$. The nominal single-scattering operator supplies the feature transform. Validation selects the surface objective $0.5L_{\mathrm{BCE}}+1.5L_{\mathrm{Dice}}+0.25L_{\mathrm{dist}}$ over weighted BCE–Dice, using positive BCE weight five and the distance normalization in Section 5.2. Checkpoints minimize validation loss on unrotated features. A common threshold maximizes mean validation F-score across the four inputs over 0.05–0.975 in steps of 0.025, giving 0.375. Inference retains three models, sixteen phases, temporal rank two and state-guided mixture weight 0.25.

A no-CSI occupancy prior averages the 64 training masks and selects a threshold from all distinct prior probabilities, including an empty prediction, on the eight validation geometries. Its selected threshold is 0.171875. All model and input choices precede testing, and comparisons use the geometry-paired bootstrap in Section 6.1. After adaptation, combined input gives F-score of 0.4120 and IoU of 0.2652 in Table 8, exceeding the prior by 0.0427 (95% CI [0.0108, 0.0772]) and 0.0360 [0.0090, 0.0661]. The squared Chamfer difference is −0.087 m^2^ ([−0.452, 0.401]), so the distance contrast remains uncertain. Direct transfer and feature calibration without retraining both produce empty predictions at the original threshold of 0.575.

Native moving-object returns are weak, with dynamic-to-calibrated-static power ratio median −33.39 dB and range [−39.82,−26.99] dB. All four inputs give F-score of 0.4120 to four decimal places; combined minus raw is $-4.36\times 10^{-6}$ (95% CI $\left[-3.58,1.98\right]\times 10^{-5}$). The native experiment shows a reconstruction gain from CSI after adaptation, with negligible differences among suppression inputs in this interference regime.

To measure how much reconstruction accuracy suppression can recover from dynamic interference, we scale the saved moving-path coefficients to dynamic-to-calibrated-static power ratios −10, 0 and +10 dB on the same 24 test geometries. These conditions are controlled amplitude tests on ray-traced components; native channels remain unscaled. All methods and intensities share the same noise realization and variance, referenced to native background-subtracted CSI, and retain the adapted decoder, feature scale, threshold, temporal rank and mixture weight. One diagnostic reference removes moving paths from the occupied-scene solve while preserving static paths and occlusion; the other traces the scene without moving objects. Both use the same noise and ground reference. Path identities construct these conditions and references, and are not algorithm inputs.

Suppression recovers progressively more reconstruction accuracy as moving-path power rises in Figure 7b,c. Combined-minus-raw F-score gains are 0.0035, 0.0232 and 0.1353 at −10, 0 and +10 dB, with 95% intervals [0.0013, 0.0059], [0.0057, 0.0437] and [0.0960, 0.1729]. At +10 dB, raw-input F-score falls to 0.2556, compared with 0.4117 when moving paths are removed. Combined suppression recovers F-score to 0.3909, raises IoU from 0.1580 to 0.2485 and reduces squared Chamfer distance from 8.409 to 1.973 m^2^. Paired changes are 0.0906 [0.0645, 0.1149] for IoU and −6.436 m^2^ [−9.578, −3.800] for Chamfer distance. Static-mean CSI NMSE decreases from 0.4248 to 0.3191.

The combined estimate integrates state-derived geometric guidance with the temporal structure of the observed CSI, retaining both information sources in the reconstruction input. State-derived delay, direction and Doppler predictions guide echo fitting, while the temporal branch estimates echo dynamics directly from CSI without using the initial target states. The combination therefore preserves geometric guidance while reducing reliance on the accuracy of the initial state estimate. Within this combination, temporal estimation provides most of the recovery in the ray-traced tests. At +10 dB, state-guided and temporal inputs give F-scores 0.3548 and 0.3946. The combination improves on state-guided input by 0.0361 (95% CI [0.0214, 0.0536]), while temporal input exceeds the combination by 0.0037 [0.0012, 0.0064]. The combination thus recovers much of the interference-induced reconstruction loss while retaining geometric guidance. Its fixed state/temporal weights of 0.25/0.75 do not adjust to the relative accuracy of the two estimates, and temporal-only suppression achieves a slightly higher F-score in this controlled condition.

### 6.8. Discussion

The experiments separate three sources of reconstruction improvement. Surface conditioning improves the spatial-prior receiver, expanded training improves the shared-CSI pipeline, and dynamic-echo suppression recovers accuracy when moving returns disrupt reconstruction. The nominal input controls and the strong-interference tests make this distinction visible: a reduction in dynamic leakage need not produce a measurable occupancy gain when raw CSI already gives similar reconstruction. Receiver calibration addresses a separate acquisition error, restoring the phase alignment needed by sensing and reconstruction.

The CSI-domain model isolates these interactions under calibrated single scattering; independent ray-tracing extends the evaluation to material-dependent reflection, path visibility and multipath after receiver adaptation. The latter uses concrete and brick buildings, moving metal objects and an ideal ground-only calibration reference, with controlled moving-path scaling for strong interference. Both studies assume known AP geometry and calibrated arrays. Nominal association uses a known count of angularly resolvable targets, with count and state-list perturbations testing departures from that setting. Measured antenna responses, time-varying background calibration and other RF impairments remain questions for measured-CSI validation. Timing accuracy and temporal identifiability define key acquisition conditions for static reconstruction.

## 7. Conclusions

Combining covariance-informed state fitting with observation-derived temporal estimation links cooperative sensing to static reconstruction through residual CSI. On 24 held-out geometries from the CSI simulator, the complete training and inference pipeline raises F-score from 0.446 to 0.480 and reduces squared Chamfer distance by 19.4%. With the decoder fixed, suppression produces a paired F-score gain of 0.0485 over raw CSI at a dynamic-to-static power ratio of +10 dB in the CSI simulator. In the controlled ray-traced moving-path test at the same power ratio, combined suppression recovers F-score from 0.256 to 0.391. Combined input also retains F-score of 0.4793 under an added velocity-error standard deviation of 8 m/s. The separate spatial-prior study shows how surface-aware refinement improves occupancy, while independent material-aware ray-tracing supports CSI-based reconstruction after receiver adaptation relative to a no-CSI prior. Together, these results establish the value of surface conditioning and training design, and identify dynamic-interference conditions in which echo suppression improves reconstruction.

## Figures and Tables

**Table 1:** CSI-domain evaluation settings and dataset partitions.

Setting	Specification
Reconstruction region/grid	20×20×9.5 m/24×24×10 cells
Shared-CSI radio and sampling	28 GHz; 64 subcarriers at 1 MHz spacing; 128 snapshots at 50 μs spacing
Shared-CSI TAP coordinates	(−18,−16,0), (18,−16,0) m
Shared-CSI RAP coordinates	(−20,14,0), (20,14,0), (0,24,2) m; calibrated 4×4 half-wavelength planar arrays
Nominal moving targets	Two resolvable targets; equal total static and dynamic powers
Evaluation SNRs	−20, −15, −10, −5, 0 and 5 dB
Shared-CSI pretraining	192 geometry seeds; 384 observations
Shared-CSI fine-tuning	448 geometry seeds; 896 observations; 447 distinct occupancy masks
Checkpoint validation	6 geometries; 36 observations
Inference development	12 geometries; 72 observations
Input/state-error test	12 geometries; 72 observations
Confirmation	24 geometries; 144 observations
Stress/calibration tests	Two separate sets, each with 12 geometries and 72 observations
Spatial-prior protocol	One moving target; 32 slow-time samples; 384 training observations
Spatial-prior evaluation	36 validation observations; 12 test geometries (72 observations); 18 independent confirmation geometries (108 observations)

Note: The radio and AP settings labeled shared-CSI apply to that protocol. The region and grid are common to both CSI-domain studies. Test, development, stress, calibration and confirmation observations use six SNRs per geometry. Training observations are counted separately from distinct geometry seeds or masks.

**Table 2:** Reference-protocol reconstruction results and principal module removals.

Configuration	F-Score ↑	Chamfer ↓	IoU ↑
		(m^2^)	
AGGRNN–MVSFNet ^†^ [12]	0.189	16.298	0.112
GMF–3D U-Net ^†^ [17]	0.237	11.415	0.142
Dual-cuboid v2 (paired)	0.412	4.165	0.264
Pretrained reference receiver	0.468	2.721	0.309
**Spatial-prior receiver**	**0.657**	**0.392**	**0.497**
Without coarse branch	0.316	6.721	0.198
Without AG enhancement	0.648	0.402	0.487
Without context refinement	0.571	0.560	0.402

Note: ↑ indicates higher is better; ↓ indicates lower is better. Bold identifies the spatial-prior receiver. Values average 72 observations from 12 base scenes. ^†^ Local adapted implementations with different training protocols. Citations identify the source ideas or backbone, not exact reproductions. The other rows describe receivers and module controls developed in this study. The pretrained reference receiver initializes the paired surface-refinement comparison and is distinct from Dual-cuboid v2. The receiver averages two widths, three seeds and three frames; v2 uses three seeds and three frames. Removal rows use frozen receiver checkpoints. Thresholds use validation only; post-processing is fixed.

**Table 3:** Shared-CSI reconstruction controls.

Input/Training Condition	F-Score ↑	Chamfer ↓	IoU ↑	ΔF [95% CI]
		(m^2^)		
No suppression	0.493	1.212	0.330	0.0021 [−0.0100, 0.0123]
Static only	0.492	1.276	0.329	0.0032 [−0.0034, 0.0107]
Position only	0.495	1.203	0.332	0.0006 [−0.0128, 0.0115]
Position + covariance	0.473	1.860	0.315	0.0224 [0.0088, 0.0365]
State-guided only	0.494	1.224	0.331	0.0016 [−0.0032, 0.0063]
State, no covariance	0.495	1.172	0.332	0.00003 [−0.00004, 0.00012]
Proposed suppression	0.495	1.172	0.332	Reference
Oracle state	0.494	1.171	0.331	0.0014 [0.0010, 0.0019]
No measurement loss	0.506	1.118	0.342	−0.0109 [−0.0248, 0.0025]

Note: ↑ indicates higher is better; ↓ indicates lower is better. Means over 72 observations from twelve test geometries. State denotes position and velocity. State-guided only removes the observation-derived estimate. Other state controls retain both estimates. Input controls share weights, phase averaging and threshold; the loss ablation is independently retrained with the same threshold. ΔF is proposed suppression minus the named row. Intervals use 20,000 paired geometry-cluster bootstrap resamples after averaging SNRs within each geometry. They are pointwise and not adjusted for multiple comparisons.

**Table 4:** Input and sensing-front-end controls with a frozen reconstruction model.

**(a) Reconstruction Input**				
Input	F-score ↑	Chamfer (m^2^) ↓	IoU ↑	Static-mean NMSE ↓
Raw	0.4583	1.9778	0.2992	0.5998
State-guided only	0.4633	2.0465	0.3036	0.5167
Observation-derived only	0.4612	2.0644	0.3021	0.5321
Combined	0.4616	2.0519	0.3023	0.5190
Oracle-state combined	0.4618	2.0487	0.3025	0.5116
**(b) Sensing Front End Followed by Combined Suppression**				
Frontend	Position RMSE (m)	Velocity RMSE (m/s)		F-score
MUSIC	5.4039	7.6466		0.4617
ESPRIT	6.6465	9.6997		0.4611
Fixed fusion	5.4374	9.3045		0.4616
Adaptive fusion	5.3575	7.2838		0.4616

Note: ↑ indicates higher is better; ↓ indicates lower is better. Bold identifies panel headings. Both panels use the same 12 stress-test geometries, six SNRs per geometry, three frozen models, 16 phases and threshold 0.575. Static-mean NMSE compares the recovered CSI mean with simulated static CSI. Oracle-state fitting receives true position and velocity but estimates waveform coefficients from mixed CSI. For combined minus raw and combined minus state-guided input, F-score differences are 0.0033 [95% CI −0.0047, 0.0120] and −0.0017 [−0.0051, 0.0014].

**Table 5:** Reconstruction F-score under scene perturbations and receiver calibration.

**(a) Synthetic Stress Tests, 12 Geometries**			
Condition	Raw	State-guided only	Combined
Nominal	0.4583	0.4633	0.4616
Receive phase SD 10 degrees	0.4583	0.4632	0.4615
Receive phase SD 30 degrees	0.4584	0.4634	0.4618
Receive timing SD 2 ns	0.4482	0.4503	0.4504
Receive timing SD 10 ns	0.3499	0.3483	0.3461
Residual slow-time CFO SD 5 Hz	0.4582	0.4629	0.4610
Residual slow-time CFO SD 20 Hz	0.4579	0.4607	0.4596
Assumed count 1, true count 2	0.4583	0.4608	0.4596
Assumed count 3, true count 2	0.4583	0.4631	0.4614
Dynamic/static ratio −10 dB	0.4700	0.4703	0.4703
Dynamic/static ratio +10 dB	0.4054	0.4461	0.4539
Velocity scale 0.1	0.2583	0.2918	0.3145
Velocity scale zero	0.2206	0.2206	0.2206
Ellipsoidal shells	0.3090	0.3054	0.3059
One estimated target removed	0.4583	0.4635	0.4598
One spurious estimated target inserted	0.4583	0.4652	0.4622
One true target, correct count supplied	0.4569	0.4623	0.4659
Three true targets, correct count supplied	0.4087	0.4249	0.4367
Additional delayed path, amplitude −10 dB	0.4447	0.4473	0.4465
**(b) Joint Receiver Mismatch, 12 Separate Calibration-Test Geometries**			
Receiver condition	Raw	State-guided only	Combined
Ideal synchronization	0.5078	0.5111	0.5115
Uncorrected offsets	0.3335	0.3341	0.3297
Estimated pilot correction	0.5073	0.5116	0.5117

Note: Bold identifies panel headings. Each panel uses six SNRs per geometry and fixed decoder settings. Panel (a) varies one condition at a time. Receiver standard deviations (SDs) precede reference subtraction. Dynamic-power and velocity interventions retain nominal noise. Panel (b) jointly applies phase, timing and CFO SDs of 30°, 10 ns and 20 Hz; calibration uses noisy reference packets with 2.5% shared airtime. Its geometries differ from panel (a), so comparisons are made within panels.

**Table 6:** Measurement-loss sensitivity and phase-averaging cost.

**(a) Fine-Tuning Loss Coefficient, Shared Initialization Within Each Seed**						
Partition	$\lambda_{\mathrm{meas}}$	F-score	CD (m^2^)	IoU	CSI residual	
Confirmation	0	0.479638	1.140058	0.318456	0.516255	
Confirmation	0.001	0.479688	1.140403	0.318515	0.547490	
Confirmation	0.01	0.479515	1.135884	0.318352	0.505935	
Confirmation	0.05	0.478798	1.145106	0.317864	0.457431	
Test	0	0.495735	1.169977	0.332638	0.547461	
Test	0.001	0.495593	1.170124	0.332511	0.578009	
Test	0.01	0.495413	1.171908	0.332354	0.531509	
Test	0.05	0.497499	1.134607	0.333965	0.472442	
**(b) Frozen-Model Phase Averaging, 24 Confirmation Geometries**						
Phases	Forwards	F-score	CD (m^2^)	IoU	Median ms	Peak MiB
1	3	0.479389	1.109302	0.318205	7.15	17.65
2	6	0.479607	1.139277	0.318432	14.17	17.67
4	12	0.479311	1.133770	0.318209	27.46	17.72
8	24	0.479532	1.135802	0.318366	46.78	17.80
16	48	0.479513	1.135910	0.318350	69.28	17.98

Note: Bold identifies panel headings. Test and confirmation partitions contain 12 and 24 geometries, respectively, with six SNRs each. Panel (a) uses three-model ensembles, 16 phases and threshold 0.575; every initial checkpoint has measurement-loss pretraining. CSI residual is the full-operator normalized squared error. Panel (b) fixes the three trained models and threshold. Timing covers device-resident phase rotation, decoder forwards and probability averaging at batch size one on an RTX 4050 Laptop GPU. Memory is peak live decoder tensor allocation, excluding operator tensors.

**Table 7:** Scene and propagation settings for independent ray-tracing.

Setting	Specification
Engine/path search	Sionna RT 2.2.0; $10^{5}$ rays per transmitter; maximum interaction depth 3; line-of-sight, specular and diffuse paths; refraction and diffraction disabled
Carrier/CSI sampling	28 GHz; 64 subcarriers at 1 MHz; 128 snapshots at 50 μs, spanning 6.35 ms
Ground plane	70×80 m concrete plane at z=−1 m
Building dimensions	Horizontal dimensions independently uniform on 2.5–4.5 m; heights 4–8 m above ground
Building centers	x∈[−6.2,−3.5] and [3.5,6.2] m; y∈[−1.5,5] m
Moving metal cuboids	Dimensions 1.8×1.5×1.7 m; centers x∈[−8,−3] and [3,8] m, y∈[−8,−4] m, z∈[3,7] m
Moving-object velocity	*x* components toward x=0 at 2–5 m/s; y∈[−1,1] and z∈[−0.2,0.2] m/s
Materials at 28 GHz	Concrete (5.24,0.626,0.2); brick (3.91,0.0406,0.2); metal $\left(1,10^{7},0.35\right)$
Transmit AP coordinates	(−18,−16,3), (18,−16,3) m; one antenna per transmitter
Receive AP coordinates	(−20,14,3), (20,14,3), (0,24,5) m
Arrays	Half-wavelength 4×4 receive arrays in the xy plane; vertically polarized isotropic elements; synthetic-array phase approximation
Geometry partitions	64 training, 8 validation and 24 test geometries; six SNRs per geometry

Note: Material entries give relative permittivity, conductivity in S/m and scattering coefficient, respectively. The two buildings are assigned concrete and brick. Native path and moving-object gains retain their ray-traced values. Background subtraction and receiver adaptation are described in the text.

**Table 8:** Reconstruction after receiver adaptation to independently ray-traced channels.

Reconstruction Input/Control	F-Score	CD (m^2^)	IoU	ΔF vs. Prior [95% CI]
Raw CSI	0.4120	1.696	0.2652	0.0427 [0.0108, 0.0772]
State-guided	0.4120	1.695	0.2652	0.0427 [0.0108, 0.0772]
Temporal	0.4120	1.696	0.2652	0.0427 [0.0108, 0.0772]
Combined	0.4120	1.696	0.2652	0.0427 [0.0108, 0.0772]
Training occupancy prior (no CSI)	0.3693	1.783	0.2292	—

Note: Values average 24 held-out geometries at 6 SNRs each. All four CSI input procedures share observations, the RT-adapted decoder and inference settings. CD is squared Chamfer distance. The occupancy-frequency prior uses training masks and a validation-selected threshold. Intervals are paired geometry-cluster percentiles from 20,000 bootstrap resamples. — denotes the reference condition.

## Data Availability

The simulation data and code supporting the findings of this study are available from the corresponding author upon request.
